# Knowledge and Perspectives of Healthcare Professionals on Point-of-Care Ultrasound in Prehospital Emergency Care in Portugal

**DOI:** 10.7759/cureus.81743

**Published:** 2025-04-05

**Authors:** Raquel Pereira, Pedro Lito, Renato Gonçalves

**Affiliations:** 1 Faculty of Health Sciences, University of Beira Interior, Covilhã, PRT; 2 Critical Care Unit, Unidade Local de Saúde da Cova da Beira, Covilhã, PRT; 3 Department of Medical Sciences, University of Beira Interior, Covilhã, PRT; 4 Department of Internal Medicine, Centro Hospitalar Cova da Beira, Covilhã, PRT

**Keywords:** emergency medicine, healthcare professionals, pocus, prehospital care, ultrasound

## Abstract

Introduction

Point-of-care ultrasound (POCUS) is an innovative and valuable tool in prehospital emergency medicine, facilitating faster and more accurate diagnoses while minimizing the need for invasive procedures. This study aimed to explore the knowledge and perspectives of prehospital emergency physicians and nurses in Portugal regarding POCUS and to assess the perceived benefits and barriers to its implementation in prehospital emergency care.

Methods

An online survey was conducted among prehospital emergency physicians and nurses in Portugal between November 2023 and January 2024. The questionnaire assessed respondents’ knowledge of POCUS, its use in both hospital and prehospital settings, and their perceptions of its relevance and feasibility in emergency care.

Results

A total of 110 responses were received. Of these, 105 (95.5%) acknowledged the benefits of POCUS. While 66 (60%) reported having knowledge of POCUS, only five (4.5%) used it in prehospital care. The primary barriers to its use were the lack of equipment and specialized training. Most respondents gained their POCUS knowledge through self-funded courses or self-learning. Nineteen (18.1%) indicated insufficient knowledge for its application. Despite these limitations, 102 (92.7%) considered the inclusion of POCUS in prehospital services to be relevant.

Conclusions

Prehospital physicians and nurses in Portugal recognize the value of POCUS, but its application is hindered by a lack of equipment and structured training. Strategic investments in training programs and portable ultrasound devices are essential to support its broader implementation in prehospital emergency care.

## Introduction

Ultrasound technology originally stemmed from research on bat echolocation and has gradually evolved into a valuable tool across many scientific disciplines, including medicine. It operates by emitting high-frequency sound waves, which are undetectable to the human ear. When these waves encounter target tissue, they produce echoes that form images. Ultrasound is widely recognized as a safe, reliable, noninvasive, and portable method for screening, diagnosing, monitoring, and treating a variety of conditions [1].

The growing interest and investment in ultrasound have spurred technological advancements that enhance both the image quality and portability of ultrasound devices, leading to their broader medical applications. Among these innovations is bedside ultrasound, also known as point-of-care ultrasound (POCUS) [2]. POCUS allows clinicians to acquire and interpret images directly at the patient’s bedside, eliminating the need for formal imaging studies, which makes it particularly useful in emergency situations [3,4].

POCUS is notable for providing immediate diagnostic answers that complement the clinical evaluation process. It is especially valuable in emergencies that require rapid diagnostic and therapeutic decisions, such as evaluating trauma victims or detecting pleural and pericardial effusions [3]. While ultrasound does not replace other diagnostic or therapeutic modalities, it can be critically important when these alternatives are either unavailable or would delay proper patient management [5].

In addition to its quick diagnostic capabilities and portability, POCUS is favored by healthcare professionals because it does not involve ionizing radiation, provides real-time imaging, and is relatively low-cost compared to other diagnostic tools [5-7].

In prehospital emergency care, healthcare professionals often work in challenging environments where diagnostic resources are limited, and decisions must be made quickly with minimal information [4]. Clinical examination is essential for prehospital diagnosis but can often be inconclusive or ambiguous. Traditional tools, such as the stethoscope, although still important, have limited utility in noisy or chaotic settings [8,9].

In this context, POCUS has proven to be a powerful and valuable tool in prehospital emergency medicine. It enhances diagnostic accuracy, accelerates the diagnostic process, and reduces complication rates when used to guide therapeutic decisions and procedures [10]. Furthermore, POCUS is a useful tool for patient triage, as it provides more accurate insights into the underlying cause of a patient’s condition, helping to determine the appropriate level of hospital care and enabling clearer communication with in-hospital teams [11].

By offering rapid imaging capabilities in the field, POCUS allows patients to be directed to the most appropriate medical facility, which may not always be the nearest one [12]. This helps to prevent geography from becoming a barrier to quality care [13] and allows for earlier initiation of treatments, particularly in cases with narrow therapeutic windows. Additionally, POCUS can help reduce unnecessary invasive procedures and can guide them more safely when required. For instance, ultrasound can aid in identifying life-threatening conditions and safely performing procedures such as thoracic drainage for a pneumothorax [11].

However, POCUS is operator-dependent, requiring adequate training to ensure both image quality and interpretation accuracy [5,14-16]. Concerns exist about the potential for POCUS to delay patient care, but studies suggest that when properly integrated, POCUS does not impede workflow [16-18]. Other barriers to its use include a lack of integration into clinical protocols and high equipment costs [12,19].

Portugal faces geographical and infrastructural disparities that affect emergency response times, particularly in remote areas. Portable ultrasound devices on emergency vehicles could help bridge these gaps [12,13].

Moreover, the structure of the Portuguese emergency medical system, coordinated by the Instituto Nacional de Emergência Médica (INEM), centralizes decision-making, which could facilitate the implementation of POCUS [20]. Additionally, Viatura Médica de Emergência e Reanimação (VMER) and the Serviço de Helicópteros de Emergência Médica (SHEM) are staffed by trained physicians and nurses, many of whom have prior hospital-based POCUS experience that can be applied to the prehospital setting [21]. Furthermore, in Portugal, knowledge of the extended focused assessment with sonography in trauma (eFAST) protocol is required for emergency medicine certification, highlighting the importance of POCUS skills among prehospital physicians [22].

A study conducted in Denmark by Pietersen et al. demonstrated that paramedics and emergency medical technicians can acquire images of sufficient quality to differentiate normal findings from pathologies, such as pleural effusions [23]. These findings underscore the potential benefits of incorporating this diagnostic modality into Portugal’s prehospital emergency units, like the Suporte Imediato de Vida (SIV), which are staffed solely by nurses and emergency technicians [24].

Although the advantages of POCUS are well recognized, no prior studies have evaluated the knowledge and use of POCUS in prehospital care in Portugal. Therefore, this research aims to assess the knowledge and perspectives of Portuguese prehospital physicians and nurses regarding POCUS, identify barriers to its implementation, and explore its potential impact on prehospital emergency care.

## Materials and methods

An extensive literature review was conducted across databases such as PubMed, SciELO, and ResearchGate to identify national and international studies on POCUS in prehospital care and to outline the primary research questions. Articles published between 2010 and 2023 in either Portuguese or English were included. Additionally, Google searches were used to clarify concepts and guidelines related to prehospital care and ultrasound. References from previously selected articles were also reviewed.

The target population comprised physicians and nurses working in prehospital emergency care in mainland Portugal. According to the 2022 activity report by the INEM, there were 2,020 physicians and 966 nurses in these services, totaling 2,986 professionals [24]. Using an online sample size calculator (Calculater.net) at a 95% confidence level and a 10% margin of error, a minimum of 94 participants was estimated. Inclusion criteria were physicians and nurses practicing prehospital emergency care in mainland Portugal who consented to participate. Exclusion criteria included those who were not physicians or nurses, were not involved in prehospital emergency care in mainland Portugal, or did not provide consent to participate.

To characterize the sample, participants were grouped by four geographical regions: North, Center, Lisbon and Tagus Valley, and South. This division was made to better align with the study’s objectives, separating Lisbon and Tagus Valley from the South (Alentejo + Algarve) due to differences in population density and health infrastructure [25].

The survey (included as an appendix) was developed based on a literature review and instruments from similar studies, tailored to the Portuguese context. It was structured to include mandatory responses for all questions. The initial section collected demographic data, followed by questions on medical/nursing specialties. The next section focused on POCUS knowledge, how it was acquired, and whether participants used POCUS in both hospital and prehospital contexts. Participants were then asked about the use of POCUS in prehospital settings, including perceived barriers. Finally, respondents provided their opinions on the adoption of POCUS in prehospital emergency teams.

Data collection took place via Google Forms (Google LLC, Mountain View, CA, United States) from November 2023 to January 2024, and duplicate responses were excluded. Descriptive analysis was performed using Microsoft Excel (Microsoft Corporation, Redmond, WA, USA) and IBM SPSS Statistics for Windows, Version 29.0.1.0 (Released 2023; IBM Corp., Armonk, NY, USA). The final total comprised 110 valid responses. Using the same online calculator, the margin of error was determined to be approximately 8.99% at a 95% confidence level for the studied population.

## Results

Sample characteristics

A total of 110 responses were collected, with 70 (63.6%) being physicians and 40 (36.4%) being nurses, aged between 27 and 62 years. Geographically, 25 (22.7%) participants worked in the North region, 66 (60%) in the Center, 14 (12.7%) in Lisbon and Tagus Valley, and five (4.5%) in the South. In terms of the type of prehospital service, six (5.5%) participants worked in SIV ambulances, 96 (87.3%) in VMER, and eight (7.3%) in the SHEM. Table 1 provides a detailed breakdown of the overall sample characteristics.

**Table 1:** Demographic information Values are presented as n (%), where n represents the absolute frequency. SHEM, Serviço de Helicópteros de Emergência Médica; SIV, Suporte Imediato de Vida; VMER, Viatura Médica de Emergência e Reanimação

Variable	Total, n (%)	Physicians, n (%)	Nurses, n (%)
Health professionals	110 (100)	70 (63.6)	40 (36.4)
Age (years)			
<30	12 (10.9)	12 (17.1)	0 (0)
30-39	41 (37.2)	35 (50)	6 (15)
40-49	32 (29)	16 (22.9)	16 (40)
50-59	22 (20)	6 (8.6)	16 (40)
≥60	3 (2.7)	1 (1.4)	2 (5)
Years in prehospital care			
<2	21 (19)	20 (28.6)	1 (2.5)
2-5	20 (18.1)	16 (22.9)	4 (10)
5-10	16 (14.5)	13 (18.6)	3 (7.5)
10-15	15 (13.6)	10 (14.2)	5 (12.5)
>15	38 (34.5)	11 (15.7)	27 (67.5)
Type of service			
SIV	6 (5.5)	0 (0)	6 (15)
VMER	96 (87.3)	64 (91.4)	32 (80)
SHEM	8 (7.3)	6 (8.6)	2 (5)
Region			
North	25 (22.7)	21 (30)	4 (10)
Center	66 (60)	35 (50)	31 (77.5)
Lisbon and Tagus Valley	14 (12.7)	10 (14.3)	4 (10)
South	5 (4.5)	4 (5.7)	1 (2.5)

Regarding years of experience in prehospital care, 38 (34.5%) had over 15 years in the field. Among physicians, 44 (62.9%) were specialists, while 26 (65%) of nurses also reported having a specialty (Table 2, Table 3).

**Table 2:** Physicians’ specialty Values are presented as n (%), where n represents the absolute frequency.

Specialty	Total, n (%)	Residents, n (%)	Specialists, n (%)
Total	70 (100)	26 (37.1)	44 (62.9)
Internal medicine	25 (35.7)	7 (10)	18 (25.7)
Intensive care	21 (30)	11 (15.7)	10 (14.3)
Anesthesiology	12 (17)	4 (5.7)	8 (11.4)
General surgery	4 (5.7)	1 (1.4)	3 (4.3)
General medicine	3 (4.3)	1 (1.4)	2 (2.9)
Cardiac surgery	1 (1.4)	1 (1.4)	0 (0)
Stomatology	1 (1.4)	0 (0)	1 (1.4)
Immunohematology	1 (1.4)	0 (0)	1 (1.4)
Nuclear medicine	1 (1.4)	0 (0)	1 (1.4)
Pulmonology	1 (1.4)	1 (1.4)	0 (0)

**Table 3:** Nurses’ specialty Values are presented as n (%), where n represents the absolute frequency.

Specialty	n (%)
Total	40 (100)
Medical-surgical	22 (55)
Rehabilitation	1 (2.5)
Maternal/obstetrics	1 (2.5)
Community health	1 (2.5)
Mental/psychiatric	1 (2.5)
Nonspecialist	14 (35)

Characterization of POCUS knowledge

**Figure 1:**
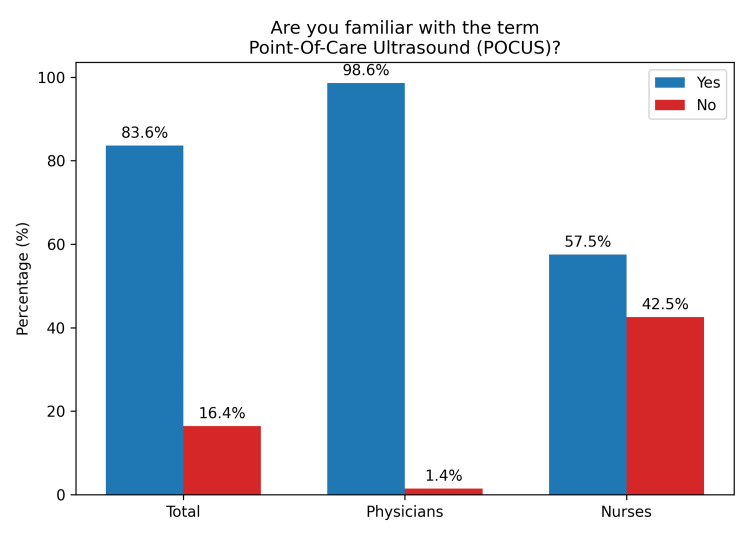
Familiarity with the term POCUS among prehospital healthcare professionals POCUS, point-of-care ultrasound

**Figure 2:**
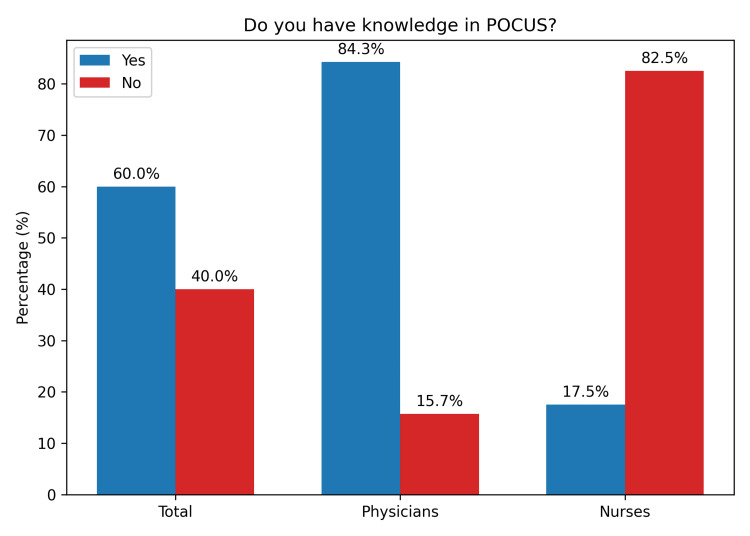
Knowledge in POCUS among prehospital healthcare professionals POCUS, point-of-care ultrasound

**Figure 3:**
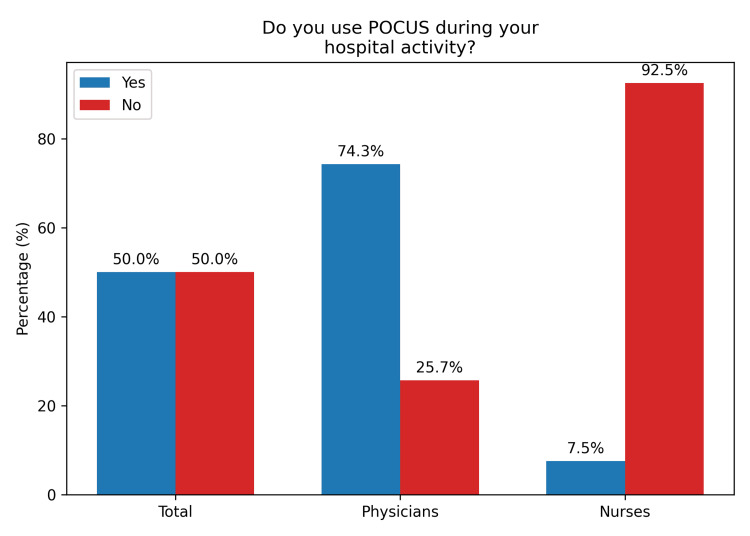
Use of POCUS in hospital practice by prehospital healthcare professionals POCUS, point-of-care ultrasound

A total of 92 (83.6%) professionals were familiar with the term “POCUS” (Figure 1). Furthermore, 66 (60%) reported having knowledge of the technique (Figure 2). Regarding its use in hospital settings, 55 (50%) indicated that they use POCUS in those environments (Figure 3).

A comparison between physician and nurse responses was conducted using p-values to assess statistical significance, with values <0.05 considered statistically significant. The analysis revealed p < 0.001 for three parameters: familiarity, knowledge, and in-hospital use of POCUS, indicating significant differences between physicians and nurses in all three domains (Table 4).

**Table 4:** Association between professional group and POCUS familiarity, knowledge, and hospital use p-Values <0.05 were considered statistically significant and were calculated using Fisher’s exact test. * These p-values indicate statistical significance. POCUS, point-of-care ultrasound

Question	p-Value
Are you familiar with the term POCUS?	<0.001*
Do you have knowledge in POCUS?	<0.001*
Do you use POCUS in your hospital activity?	<0.001*

**Figure 4:**
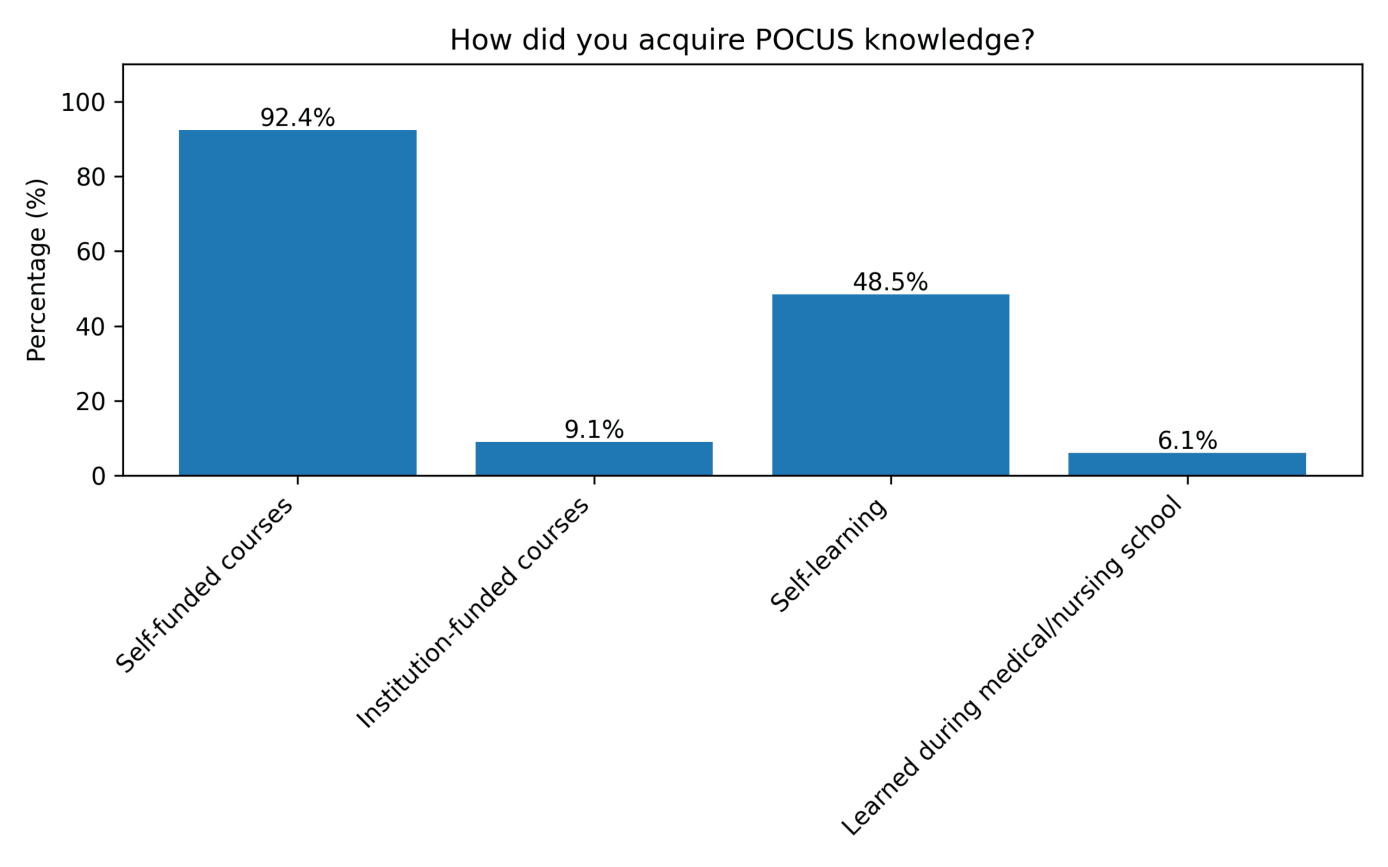
Methods of acquiring knowledge in POCUS Participants could choose more than one option. POCUS, point-of-care ultrasound

Participants who reported having knowledge of POCUS were asked how they had acquired it. A total of 61 (92.4%) indicated attending self-funded courses, 32 (48.5%) were self-taught, six (9.1%) received institution-funded training, and four (6.1%) gained knowledge during formal academic education (Figure 4).

Opinions on POCUS use in prehospital care

**Figure 5:**
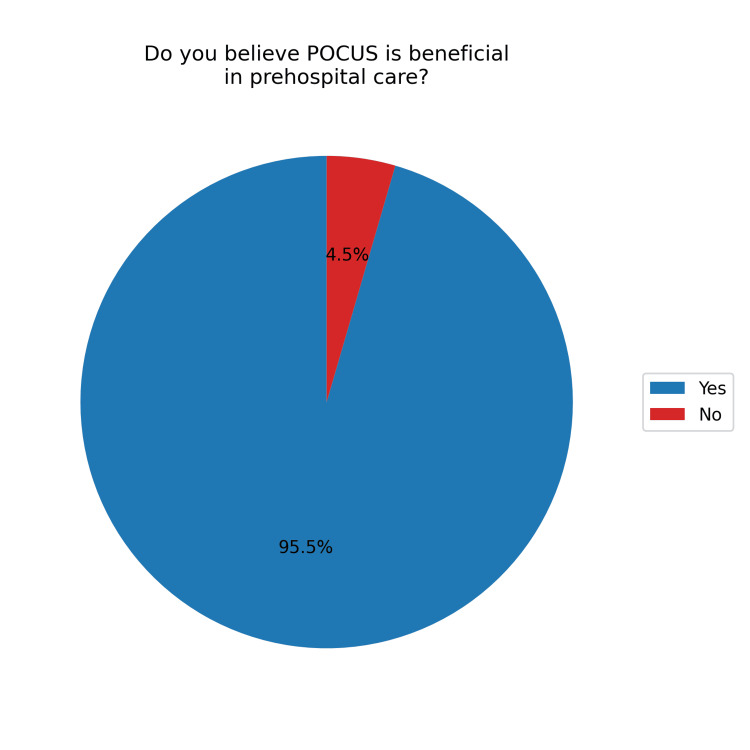
Perception of the benefits of POCUS in prehospital care POCUS, point-of-care ultrasound

**Figure 6:**
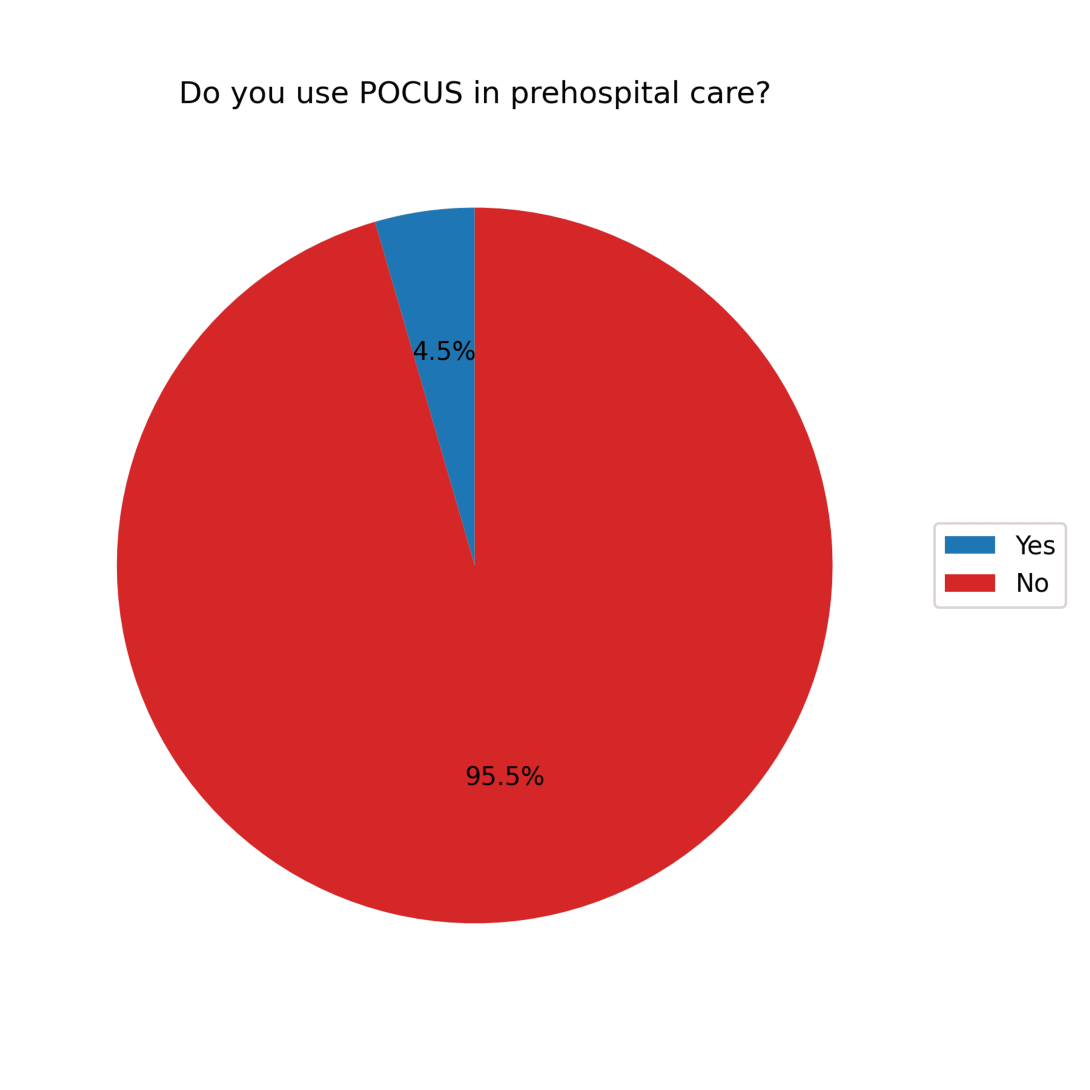
Use of POCUS in prehospital care POCUS, point-of-care ultrasound

**Figure 7:**
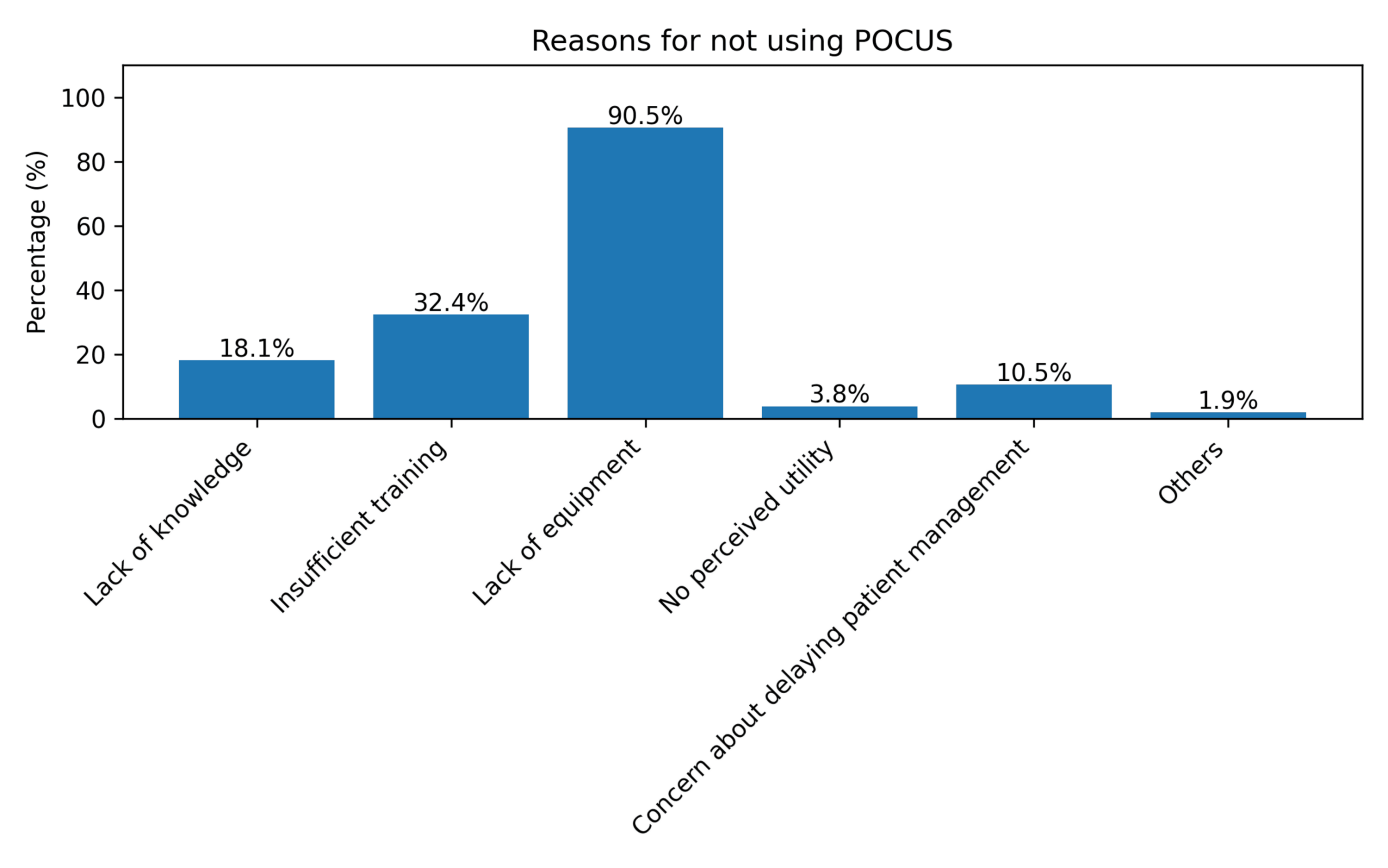
Reasons for not using POCUS in prehospital care Participants could choose more than one option. POCUS, point-of-care ultrasound

Regarding the use of POCUS in prehospital care, 105 (95.5%) respondents believed it would be beneficial (Figure 5). However, only five (4.5%) reported actually using POCUS in this setting (Figure 6). The main barrier cited was a lack of equipment, reported by 95 (90.5%) participants. Additionally, 34 (32.4%) felt they had insufficient training, and 19 (18.1%) cited a lack of knowledge. Other concerns included 11 (10.5%) participants fearing that POCUS might delay care and four (3.8%) believing it would not be helpful in prehospital settings (Figure 7).

Characterizing POCUS use in prehospital care

As only five (4.5%) healthcare professionals reported using POCUS in the prehospital setting, the sample size was insufficient to form a robust characterization of its usage in this context.

Perspectives on POCUS in prehospital care

**Figure 8:**
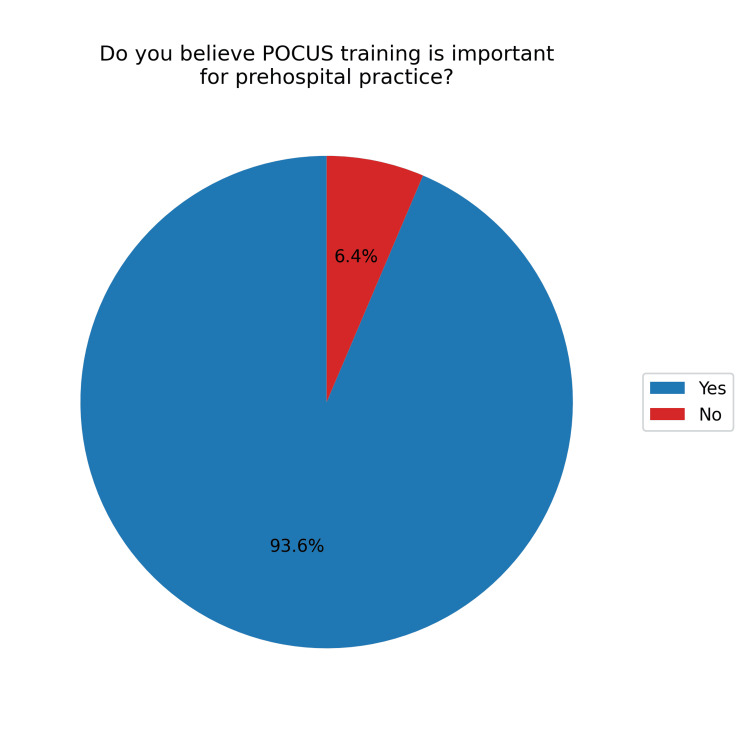
Perception of the importance of POCUS training for prehospital practice POCUS, point-of-care ultrasound

**Figure 9:**
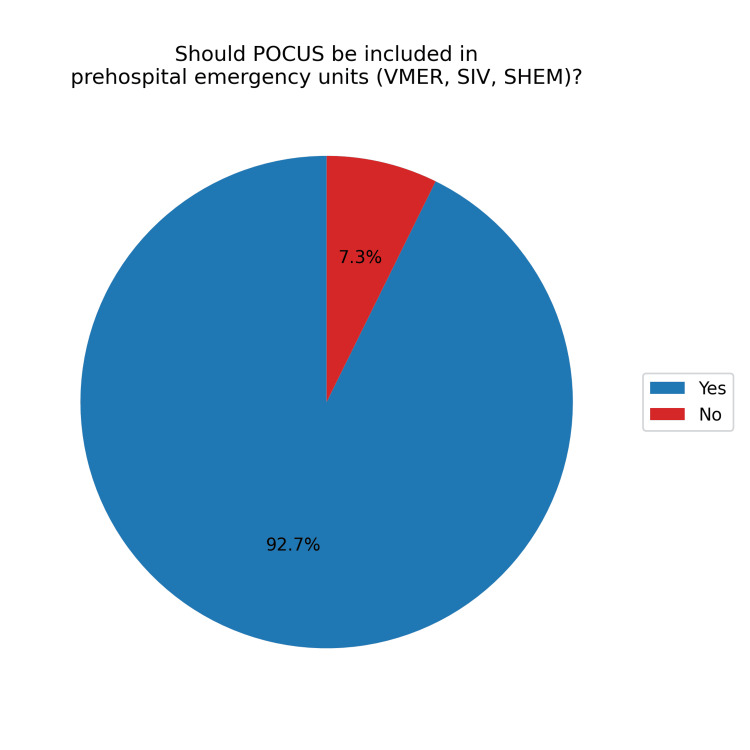
Percentage of participants who believe POCUS should be included in prehospital emergency services POCUS, point-of-care ultrasound

Looking toward the future, 103 (93.6%) believed that training in POCUS for prehospital work is important (Figure 8), and 102 (92.7%) felt that POCUS should be included in emergency units such as VMER, SIV, and SHEM (Figure 9).

## Discussion

This study offers the first national insight into the perspectives of Portuguese prehospital professionals on POCUS. While most participants are familiar with the technology and recognize its benefits, its use remains limited. Training is primarily acquired independently, indicating a need for institutional support.

Physicians reported greater knowledge and use of POCUS compared to nurses. However, past studies, such as that by Pietersen et al., suggest that with appropriate training, nonphysician professionals (e.g., nurses) can also effectively use POCUS. This is particularly relevant in Portugal, where some emergency units are staffed exclusively by nurses and emergency technicians [23].

Although 105 (95.5%) of the participants reported using POCUS in hospital practice, only five (4.5%) applied it in the prehospital setting. The primary limitation was the lack of portable ultrasound equipment. Similar findings were observed in a North American study by Taylor et al., where among 222 EMS professionals surveyed (predominantly paramedics, with only 2.3% being physicians), just nine (4.1%) applied POCUS, primarily due to equipment costs and inadequate training [26]. In France, Bobbia et al. found that only 25 out of 278 prehospital services had ultrasound devices available, corresponding to a 9% availability rate, which further underscores this gap [27].

In contrast, the United Kingdom has reported higher rates of POCUS adoption in prehospital settings, particularly among air ambulance units. This demonstrates that POCUS can be successfully integrated into prehospital emergency care [28].

Based on the findings of this study and relevant literature, it would be beneficial to develop and offer structured POCUS training programs specifically tailored to the prehospital context. These programs should ideally include basic protocols (such as eFAST), hands-on workshops, and ongoing competency assessments [5,14].

Despite limitations in equipment and training, this study found strong support among Portuguese prehospital healthcare professionals for the adoption of POCUS. Portable ultrasound technology has advanced significantly, with devices becoming more portable, durable, and user-friendly, making them suitable for use in field conditions. However, their cost remains a substantial investment [11]. Cost-benefit analyses are needed to assess the purchase and maintenance of ultrasound devices against potential reductions in morbidity, mortality, and other healthcare costs.

To facilitate the adoption of POCUS, implementation strategies should include its integration into national emergency medical protocols and the development of standardized operating procedures [12]. The centralized structure of the Portuguese emergency medical system presents a unique opportunity to streamline and standardize such measures across the country [21].

## Conclusions

Prehospital emergency physicians and nurses in Portugal recognize the value of POCUS and its potential to improve care. However, its implementation is hindered by limited access to equipment and insufficient training. Strategic investments in portable devices and the development of structured training programs are essential to overcoming these barriers.

Future research should focus on conducting a cost-benefit analysis of nationwide POCUS implementation, considering its potential to reduce morbidity, mortality, and healthcare disparities.

## Appendices

Survey

My name is Raquel Pereira, and I am a 5th-year medical student at the University of Beira Interior. As part of my master’s dissertation and under the supervision of Dr. Pedro Lito and Dr. Renato Gonçalves, I am conducting research on the application of Point-of-Care Ultrasound (POCUS). POCUS has proven to be a valuable tool in emergency medicine, improving diagnostic accuracy and accelerating the diagnostic process. Portugal has favorable conditions for its implementation, including a centralized emergency medical system and the Emergency Medicine competence recognized by the Portuguese Medical Association. However, there is currently no literature describing the use of this tool in Portugal or the opinions of healthcare professionals regarding it. This survey is therefore directed at physicians and nurses working in VMER, SIV, and SHEM units. Its objective is to assess the current knowledge of prehospital healthcare professionals in Portugal regarding POCUS, as well as its implementation, usage, perceived benefits, and barriers. This survey aims to contribute to the understanding of POCUS use in the prehospital setting and to help overcome identified obstacles in order to improve clinical outcomes. Your participation in this study is voluntary and anonymous. All collected data will be treated confidentially. Thank you for your contribution.

* Indicates a required question

Having read this information, I declare that I agree to participate in this study*

- Yes - No

Demographic Information

Age* ___________

How many years have you worked in prehospital emergency care?*

- Less than 2 years - 2 to 5 years - 5 to 10 years - 10 to 15 years - More than 15 years

In which type of prehospital emergency unit do you primarily work?*

- Immediate Life Support Ambulance (SIV) - Medical Emergency and Resuscitation Vehicle (VMER) - Emergency Medical Helicopter Service (SHEM)

In which region do you primarily work?*

- North - Center - Lisbon and Tagus Valley - South

Professional role*

- Physician - Nurse

Medical Specialty

Are you a resident or a specialist? - Resident - Specialist

What is your specialty? 

- Pathological Anatomy

- Anesthesiology

 - Angiology and Vascular Surgery

- Cardiology

- Pediatric Cardiology

- Cardiac Surgery

- Cardiothoracic Surgery

- General Surgery

- Maxillofacial Surgery

- Pediatric Surgery

- Plastic, Reconstructive and Aesthetic Surgery

- Thoracic Surgery

- Dermatovenereology

- Infectious Diseases

- Endocrinology and Nutrition

- Stomatology

- Clinical Pharmacology

- Gastroenterology

- Medical Genetics

- Obstetrics and Gynecology

- Clinical Hematology

- Immunoallergology

- Immunohemotherapy

- Sports Medicine

- Occupational Medicine

- Physical and Rehabilitation Medicine

- General and Family Medicine

- Intensive Care Medicine

- Internal Medicine

- Forensic Medicine

- Nuclear Medicine

- Tropical Medicine

- Nephrology

- Neurosurgery

- Neurology

- Neuroradiology

- Ophthalmology

- Medical Oncology

- Orthopedics

- Otorhinolaryngology

- Clinical Pathology

- Pediatrics

- Pulmonology

- Psychiatry

- Child and Adolescent Psychiatry

- Radiology

- Radiation Oncology

- Rheumatology

- Public Health

- Urology

Nursing Specialty

Are you a specialist nurse? - Yes - No

If yes, what is your specialty/work area? _____________

POCUS Knowledge

Are you familiar with the term Point-of-Care Ultrasound (POCUS)?* - Yes - No

Do you have knowledge of POCUS?* - Yes - No

If yes, how did you acquire this knowledge? (Select all that apply): - Self-funded courses or training - Institution-funded training - Self-directed learning - Training provided by my medical/nursing school - Other:______________

Do you use POCUS in a hospital setting?* - Yes - No

Opinion on POCUS in Prehospital Emergency Care

In your opinion, can POCUS benefit the management of critical patients in prehospital settings?* - Yes - No

Do you use POCUS during your prehospital emergency activity?* - Yes - No

If not, what are the reasons? (Select all that apply): - Lack of knowledge - Insufficient training - Lack of equipment - Do not consider it useful - It delays patient management - Other: ______________

POCUS Usage in Prehospital Emergency Care

How frequently do you use POCUS in prehospital care? - Infrequently - Frequently - Very frequently

In what situations do you use POCUS in prehospital care? (Select all that apply): - For diagnostic support - To guide procedures - For therapeutic purposes and monitoring - During cardiopulmonary arrest management - Other:_________________

Do you believe your clinical outcomes improve when using POCUS? - Yes - No

Do you feel more confident in decision-making when using POCUS? - Yes - No

Have you noticed improved outcomes since incorporating POCUS into your skill set? - Yes - No

What do you consider to be the benefits of POCUS use? (Select all that apply): - Reduces clinical intervention time - Facilitates interventional procedures - Helps monitor the patient - Speeds up clinical diagnosis - Guides and improves clinical management - Other:_________________

Was the POCUS equipment you use personally funded or institutionally provided? - Personally funded - Provided by the employing institution - Other:___________

Personal Perspective

Do you consider POCUS knowledge important for prehospital care?* - Yes - No

If feasible, do you believe this tool should be included in VMER/SHEM/SIV units?* - Yes - No

## References

[1] Kaproth-Joslin KA, Nicola R, Dogra VS (2015). The history of US: from bats and boats to the bedside and beyond: RSNA centennial article. Radiographics.

[2] Choi W, Cho YS, Ha YR (2023). Role of point-of-care ultrasound in critical care and emergency medicine: update and future perspective. Clin Exp Emerg Med.

[3] Rice JA, Brewer J, Speaks T, Choi C, Lahsaei P, Romito BT (2021). The POCUS consult: how point of care ultrasound helps guide medical decision making. Int J Gen Med.

[4] Amaral CB, Ralston DC, Becker TK (2020). Prehospital point-of-care ultrasound: a transformative technology. SAGE Open Med.

[5] Shrestha R, Blank W, Shrestha AP, Pradhan A (2020). Evaluation of interdisciplinary emergency ultrasound workshop for primary care physicians in Nepal. Open Access Emerg Med.

[6] Hansen W, Mitchell CE, Bhattarai B, Ayutyanont N, Stowell JR (2017). Perception of point-of-care ultrasound performed by emergency medicine physicians. J Clin Ultrasound.

[7] Haydar SA, Moore ET, Higgins GL 3rd, Irish CB, Owens WB, Strout TD (2012). Effect of bedside ultrasonography on the certainty of physician clinical decisionmaking for septic patients in the emergency department. Ann Emerg Med.

[8] Bastos MG, Vieira AL, Nascimento MM, Barros E, Pazeli JM Jr, Kirsztajn GM (2021). Point-of-care ultrasonography in nephrology: a cross-sectional national survey among Brazilian nephrologists. J Bras Nefrol.

[9] Griffiths E (2021). Helicopter emergency medical services use of thoracic point of care ultrasound for pneumothorax: a systematic review and meta-analysis. Scand J Trauma Resusc Emerg Med.

[10] Reynolds TA, Amato S, Kulola I, Chen CJ, Mfinanga J, Sawe HR (2018). Impact of point-of-care ultrasound on clinical decision-making at an urban emergency department in Tanzania. PLoS ONE.

[11] (2024). Portable ultrasound devices in the pre-hospital setting: a review of clinical and cost-effectiveness and guidelines [Internet]. https://pubmed.ncbi.nlm.nih.gov/26985544/.

[12] Nelson BP, Sanghvi A (2016). Out of hospital point of care ultrasound: current use models and future directions. Eur J Trauma Emerg Surg.

[13] Lobo MJ, Tavares SC, Pereira de Almeida RP (2022). Point of care prehospital ultrasound in basic emergency services in Portugal. Health Sci Rep.

[14] Schnittke N, Damewood S (2019). Identifying and overcoming barriers to resident use of point-of-care ultrasound. West J Emerg Med.

[15] Micks T, Sue K, Rogers P (2016). Barriers to point-of-care ultrasound use in rural emergency departments. CJEM.

[16] El Sayed MJ, Zaghrini E (2013). Prehospital emergency ultrasound: a review of current clinical applications, challenges, and future implications. Emerg Med Int.

[17] Scharonow M, Weilbach C (2018). Prehospital point-of-care emergency ultrasound: a cohort study. Scand J Trauma Resusc Emerg Med.

[18] Hoyer HX, Vogl S, Schiemann U, Haug A, Stolpe E, Michalski T (2010). Prehospital ultrasound in emergency medicine: incidence, feasibility, indications and diagnoses. Eur J Emerg Med.

[19] Rimbaut E, Verhoeven E, De Smedt L (2022). Overview of current implementation and limitations of point-of-care ultrasound in the emergency department: a nationwide survey in Belgium. Acta Clin Belg.

[20] SIEM SISTEMA INTEGRADO (2024). Integrated Medical Emergency System (SIEM) [in Portuguese]. https://www.inem.pt/2017/09/21/sistema-integrado-de-emergencia-medica-sabe-o-que-e/.

[21] (2024). Best practice guidelines in trauma [in Portuguese]. https://www2.ordemdosmedicos.pt/normas-de-boa-pratica-em-trauma/.

[22] (2024). Competence in medical emergency [in Portuguese]. https://ordemdosmedicos.pt/competencia-em-emergencia-medica/.

[23] Pietersen PI, Mikkelsen S, Lassen AT (2021). Quality of focused thoracic ultrasound performed by emergency medical technicians and paramedics in a prehospital setting: a feasibility study. Scand J Trauma Resusc Emerg Med.

[24] (2024). Activity report of emergency medical services [in Portuguese]. https://www.inem.pt/wp-content/uploads/2023/07/Relatorio-Meios-de-Emergencia-Medica-2022_VFF_17072023.pdf.

[25] National Institute of Statistics (INE), Statistics Portugal (2024). Health Statistics 2022 [in Portuguese]. https://www.ine.pt/xportal/xmain?xpid=INE&xpgid=ine_publicacoes&PUBLICACOESpub_boui=439489924&PUBLICACOESmodo=2&xlang=pt.

[26] Taylor J, McLaughlin K, McRae A, Lang E, Anton A (2014). Use of prehospital ultrasound in North America: a survey of emergency medical services medical directors. BMC Emerg Med.

[27] Bobbia X, Hansel N, Muller L (2014). Availability and practice of bedside ultrasonography in emergency rooms and prehospital setting: a French survey. Ann Fr Anesth Reanim.

[28] Naeem S, Edmunds C, Hirst T (2022). A national survey of prehospital care services of United Kingdom for use, governance and perception of prehospital point of care ultrasound. POCUS J.

